# Safety and efficacy of remimazolam besylate in patients undergoing colonoscopy: A multicentre, single-blind, randomized, controlled, phase Ⅲ trial

**DOI:** 10.3389/fphar.2022.900723

**Published:** 2022-10-05

**Authors:** Ximei Wang, Xiaolei Hu, Nianyue Bai, Lie Li, Min Zhang, Zhigang Cheng, Qulian Guo

**Affiliations:** ^1^ Department of Anesthesiology, Xiangya Hospital, Central South University, Changsha, Hunan, China; ^2^ National Institution of Drug Clinical Trial, Xiangya Hospital, Central South University, Changsha, Hunan, China; ^3^ Yichang Humanwell Pharmaceutical Co., Ltd., Yichang, Hubei, China; ^4^ Humanwell Healthcare (Group) Co., Ltd., Wuhan, Hubei, China; ^5^ Department of Clinical Research Center, Yichang Humanwell Pharmaceutical Co., Ltd., Yichang, Hubei, China

**Keywords:** remimazolam besylate, propofol, sedation, adverse, events

## Abstract

**Study objective:** The objective of the study was to evaluate the safety and efficacy of remimazolam besylate versus propofol injection in patients undergoing colonoscopy.

**Design:** A multicenter, randomized, non-inferiority, single-blind, parallel-controlled clinical trial.

**Setting:** Operating room.

**Patients:** Patients aged 18–65 years (American Society of Anesthesiologists [ASA] classification I-III) undergoing a diagnostic or therapeutic colonoscopy.

**Interventions:** Patients were administered intravenous injection of remimazolam besylate or propofol (active comparator) for sedation.

**Measurements:** Modified Observer’s Assessment of Alertness/Sedation [MOAA/S] scores of the included patients were assessed before dosing, 1, 1.5, 2, 2.5, and 3 min after the start of dosing, and then every 1 min until the MOAA/S score reached 5 on three consecutive occasions.

**Main Results:** A total of 360 patients received remimazolam and 120 patients received propofol. The incidence of adverse events (67.8% vs. 84.2%, *p* = 0.001) was significantly lower in patients administered remimazolam compared to propofol. There was no significant difference in sedation success rates (full analysis set [FAS]: 98.9% vs. 99.2%; remimazolam vs. propofol). Remimazolam had a significantly longer onset of action, but the difference was not considered clinically significant (1.45 min vs. 1.24 min, remimazolam vs. propofol). Propofol achieved a deeper level of sedation (mean MOAA/S score 0.5 vs. 0.2; remimazolam vs. propofol). Mean time to discharge after the end of the last administration of study drug (20.3 vs. 21.8 min, *p* = 0.020) and incidence of injection pain was significantly lower in patients administered remimazolam (2.3% vs. 35.3%, *p* < 0.0001). Incidence of oxygen desaturation was significantly higher in patients administered propofol compared to patients administered remimazolam (6.7% vs. 1.1%, *p* = 0.001). Similarly, incidence of hypotension was more frequent in patients administered propofol compared to patients administered remimazolam (29.2% vs. 10.6%, *p* < 0.0001).

**Conclusion:** Remimazolam besylate had a better safety and tolerability profile and similar sedative efficacy to propofol in patients undergoing a diagnostic or therapeutic colonoscopy in China, suggesting that remimazolam besylate has potential as a sedative agent for colonoscopy.

## Introduction

Nausea, vomiting, and abdominal pain may develop in patients undergoing traditional colonoscopy, gastroscopy, or other gastrointestinal endoscopy procedures (Soweid et al., 2011; Li et al., 2019). These symptoms may prolong the procedure and cause patient discomfort and anxiety. The use of analgesia and sedation for colonoscopy, gastroscopy, or other gastrointestinal endoscopy procedures can improve patient satisfaction and cooperation with the procedure and the quality of the examination (Meining et al., 2007).

Intravenous sedative drugs commonly used in clinical practice include propofol, midazolam, and dexmedetomidine (Martin et al., 2003; Eger, 2004; Inadomi et al., 2010; Keating, 2015). Propofol is short-acting and has a rapid onset of action (Dunn et al., 2007; Black et al., 2013). Although propofol is an effective anesthetic it has been associated with several adverse events. Propofol can affect the cardiovascular system and cause oxygen desaturation, such that the involvement of an anesthesiologist is recommended for the care of every patient undergoing propofol anesthesia (Wesolowski et al., 2016). In addition, propofol may cause pain on injection (Dedic et al., 2010; Euasobhon et al., 2016), exhibit nonlinear kinetics (Wesolowski et al., 2016), and some formulations carry the risk of bacterial contamination (McHugh and Roper, 1995; Soong, 1999). Midazolam, the first water-soluble benzodiazepine drug, has been widely used in sedation and induction of general anesthesia (Reves et al., 1985). However, midazolam is long-acting and recovery is delayed (Black et al., 2013). Midazolam is metabolized by cytochrome P450 in the liver to an active metabolite; therefore, hepatic function can affect midazolam clearance (Zaporowska-Stachowiak et al., 2019). Dexmedetomidine is a safe and efficacious sedative with a rapid onset of action. However, dexmedetomidine has been associated with hemodynamic instability (hypertension, bradycardia) (Ice et al., 2016), requires a complex dosing regimen based on body weight and response (Weerink et al., 2017; Inatomi et al., 2018), has a slow recovery profile (Schacherer et al., 2019), and there are no effective dexmedetomidine reversal agents.

Remimazolam is a novel ultra-short-acting benzodiazepine that has sedative/narcotic effects. Remimazolam acts on γ-aminobutyric acid A (GABA_A_) receptors to inhibit neural activity by increasing chloride influx and hyperpolarizing postsynaptic neurons (Kilpatrick et al., 2007). Remimazolam besylate is water soluble, has a short elimination half-life, is not subject to P450 enzyme metabolism, and is eliminated as inactive metabolites. Remimazolam besylate may be used for sedation, induction and maintenance of general anesthesia, and sedation during diagnostic or therapeutic procedures in the intensive care unit (ICU). Remimazolam tosylate was shown to be non-inferior in sedation efficacy and safer than propofol in patients undergoing colonoscopy in China (Chen et al., 2020). Based on these data and accumulating evidence from other clinical studies (Antonik et al., 2012; Borkett et al., 2015; Pambianco et al., 2016; Rex et al., 2018), we hypothesized that remimazolam besylate is a safe and effective sedative for use in patients undergoing colonoscopy in China. This Phase Ⅲ clinical trial evaluated the safety and efficacy of remimazolam besylate vs. propofol (active comparator) injection for sedation in patients undergoing a diagnostic or therapeutic colonoscopy in China.

## Materials and methods

### Patient population

This was a multicenter, randomized, single-blind, parallel-controlled clinical trial evaluating the safety and efficacy of remimazolam besylate vs. propofol medium/long-chain fat emulsion (active comparator) injection for sedation in patients undergoing a diagnostic or therapeutic colonoscopy in China. All participating centers were tertiary hospitals with expertise in conducting clinical trials. Standardized training was provided to researchers in each center before trial initiation. Detailed inclusion and exclusion criteria are shown in Table 1. Key inclusion criteria were: 1) scheduled to undergo a diagnostic or therapeutic colonoscopy; 2) aged 18–65 years; 3) body mass index (BMI) 18–28 kg/m^2^; 4) American Society of Anesthesiologists (ASA) physical status classification system risk class I∼III. Exclusion criteria were: 1) allergy or contraindication to benzodiazepines, opioids, propofol, flumazenil, naloxone or their components; 2) severe respiratory disease; 3) abnormal liver or kidney function; 4) difficult airway; 5) craniocerebral injury; 6) mental illness or cognitive impairment; 7) resting ECG heart rate <50 beats/min, QTc: male ≥470 ms female ≥480 ms, third degree atrioventricular block, severe arrhythmia and/or moderate to severe heart valve disease.

**TABLE 1:** Inclusion and Exclusion criteria.

Inclusion criteria
• Age 18–65 years
• Body mass index (BMI) 18–28 kg/m^2^
• American Society of Anesthesiologists (ASA) classification I-III
• Undergoing a diagnostic or therapeutic colonoscopy
• Voluntarily provided written informed consent
Exclusion criteria
• Allergy or contraindication to benzodiazepines, opioids, propofol, flumazenil, naloxone or their components
• At screening, resting ECG heart rate <50 beats/min, QTc: male ≥470 ms female ≥480 ms, third degree atrioventricular block, severe arrhythmia and/or moderate to severe heart valve disease; and/or history of acute heart failure, unstable angina and/or myocardial infarction within 6 months prior to screening
• Severe respiratory disease
• Poor blood pressure control (systolic blood pressure [SBP]≥160 mmHg or ≤90 mmHg) and not receiving regular antihypertensive therapy
• Abnormal liver or kidney function: alanine aminotransferase (ALT) and/or aspartate transaminase (AST) 2.5 X the upper limit of the medical reference value, urea or urea nitrogen ≥1.5X upper limit of normal (ULN) and/or blood creatinine > ULN
• Difficult airway (modified Mallampati classification ≥ III)
• Craniocerebral injury, possible intracranial hypertension and/or cerebral aneurysm; and/or history of cerebrovascular accident or central nervous system disease

### Study design

The study included a screening visit (Visit 1; Day -7 to −1), the procedure (Visit 2; Day 1) and an outpatient follow-up visit for safety assessments (Visit 3; Day 2–5). Following successful screening and consent, patients were randomized 3:1 to remimazolam besylate or propofol as the active comparator.

All patients completed bowel preparation before colonoscopy. Immediately prior to the procedure, patients were provided inhaled oxygen (flow rate 2–4 L/min), they were fully alert (three consecutive Modified Observer’s Assessment of Alertness/Sedation [MOAA/S] scores of 5 (Chernik et al., 1990); painful stimulation was through the trapezius squeeze, a score of 0 meant no response), and patients were pretreated with analgesia (fentanyl 50 µg) by intravenous injection.

According to their treatment allocation, patients were administered an intravenous injection of remimazolam besylate 7 mg (Yichang Humanwell Pharmaceutical CO., Ltd., China) or propofol 1.5 mg/kg over 1 min (±5 s) (Fresenius Kabi Austria GmbH, Austria); the start of study drug administration was t = 0. Colonoscopy was initiated when adequate sedation (MOAA/S ≤ 3) was achieved. Top-up doses of remimazolam besylate 2.5 mg or propofol 0.5 mg/kg were administered to patients with an MOAA/S score ≥4 at 3 min. During induction of sedation, a maximum of 4 top-up doses of remimazolam besylate or propofol were administered at ≥2 min intervals. To maintain sedation while performing the diagnostic or therapeutic colonoscopy, a maximum of 5 doses of remimazolam besylate 2.5 mg or propofol 0.5 mg/kg were administered during any 15 min interval. Sedation failure was defined as a requirement of >5 doses remimazolam besylate 2.5 mg or propofol 0.5 mg/kg and the use of rescue agents (propofol or propofol medium/long chain fat emulsion injection) to complete the procedure.

The clinical trial was registered at http://www.chinadrugtrials.org.cn/(registration No. CTR20180510, registration date: Sept.7, 2018) and was conducted in accordance with the Declaration of Helsinki and standards of Good Clinical Practice. The clinical trial protocol and all amendments were approved by the appropriate ethics body at each participating institution. All patients provided written informed consent before enrolment.

### Randomization and blinding

Patients were randomized using stratified permuted block randomization *via* the DAS Interactive Web Response System (IWRS) (Beijing Bozhiyin Technology Co., Ltd.). Patients were stratified by center, and permuted block randomization was used for each stratum. Block randomization was by a computer-generated random number list (SAS software). Study patients were blinded to the randomization codes. Investigators and study site personnel were unblinded to the treatment allocation as they could easily differentiate the sedative agents based on their appearance, and knowledge of the treatment assignment was required in case of a safety emergency.

### Safety and efficacy assessments

Safety assessments included adverse events, vital signs, physical examinations, laboratory examinations (routine hematology, blood biochemistry, urinalysis), twelve-lead electrocardiograms, and study discontinuation due to safety and/or tolerability concerns. Clinically important adverse events of special interest included 1) incidence of oxygen desaturation (oxygen saturation <90%) during sedation; 2) incidence of hypotension (SBP ≤80mmHg) during sedation; 3) incidence of hypotension necessitating intervention (SBP ≤80mmHg and the fall was 30% below baseline) during sedation; intervention for hypotension included treatment with vasopressor drugs from initial administration of trial drug to fully alert; 4) treatment emergent adverse events, which were recorded throughout the trial period. The severity of treatment emergent adverse events was assessed according to the National Cancer Institute Common Terminology Criteria for Adverse Events (NCI-CTCAE) version 4.0 (Trotti et al., 2003), where Grade 1 to 4 events were classified as mild, moderate, severe, and life-threatening or disabling, respectively.

The primary efficacy outcome measure was the sedation success rate. Procedure success was defined as completion of diagnostic or therapeutic colonoscopy with ≤5 doses (initial dose plus 4 top-up doses) of remimazolam besylate or propofol administered during any 15 min interval and no use of rescue agents. Secondary efficacy outcome measures were: 1) mean time from the start of study drug administration (t = 0) to first MOAA/S score ≤3; 2) mean time from the end of the last study drug administration to fully alert (first occurrence of three consecutive MOAA/S scores of 5); 3) mean time from the end of the last study drug administration to discharge; 4) change in MOAA/S score over time; 5) evaluation of injection pain. Anesthesiologists oversaw all procedures and performed the safety and efficacy assessments. First exposure time, total exposure time and total exposure were also used for evaluating the efficacy of the study drugs. First exposure time = end time of first dose - start time of first dose; Total exposure time = (end time of first dose - start time of first dose) + ∑ (end time of additional dose-start time of additional dose); Total exposure = first exposure + additional total exposure.

### Sample size calculation

Sample size calculation was based on a Phase II clinical trial, which revealed a sedation success rate of 98% for both study drugs. PASS 14.0 software was used to estimate the sample size at 247 patients administered remimazolam besylate and 82 patients administered propofol (3:1), assuming a noninferiority margin of 5%, *α* = 0.025, and *β* = 0.2 for a one-sided test. Taking into account regulatory requirements and dropout, the present study planned to enroll 480 patients (360 patients administered remimazolam besylate and 120 patients administered propofol).

### Statistical analysis

Statistical analysis was performed with SAS software v9.4. The full analysis set (FAS) included patients who were administered at least one dose of study drug, and had baseline demographic and clinical data and at least one efficacy evaluation after administration in accordance with intention-to-treat (ITT) principles. The per-protocol set (PPS) included patients in the FAS with no protocol deviations. The safety set (SS) included patients who had received at least one dose of study drug and had at least one safety evaluation. Descriptive statistics were used to evaluate all data, including demographics and safety and efficacy outcomes. Non-inferiority was to be claimed if the two-sided 95% confidence interval of the primary efficacy outcome difference between remimazolam besylate and propofol was within a predetermined noninferiority margin (-5%). Within-group comparisons were performed using the t test or Wilcoxon rank sum test, depending on whether data were normally or non-normally distributed. All statistical tests were two-sided, and *p* ≤ 0.05 was considered statistically significant.

## Results

### Patient population

**FIGURE 1:**
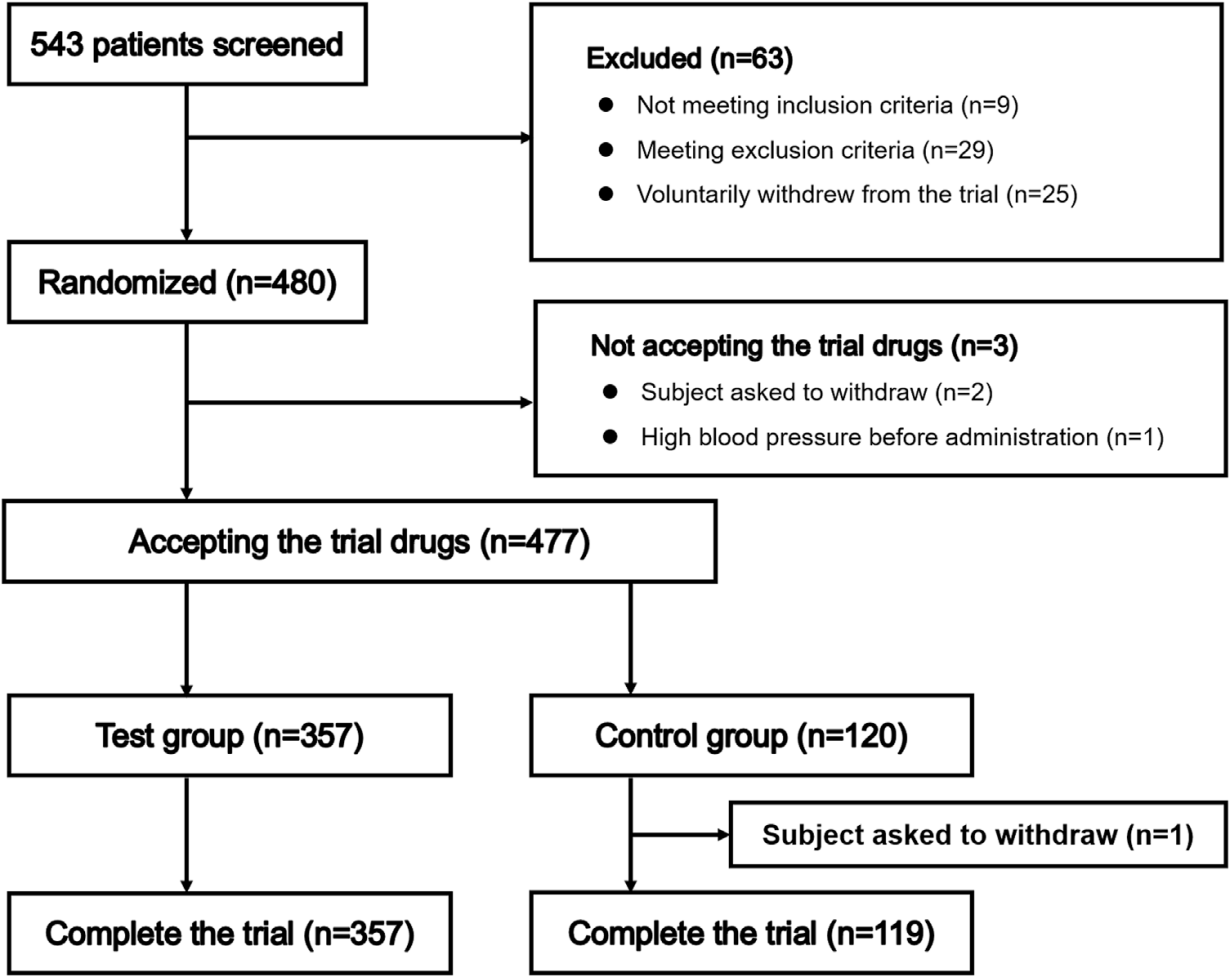
Patient attrition.

Patient selection is summarized in Figure 1. A total of 543 patients entered pre-trial screening between April 2018 and July 2018, and 480 patients were enrolled, including 360 patients randomized to receive remimazolam besylate and 120 patients randomized to receive propofol. Three patients did not receive study drug as they had high blood pressure prior to administration or withdrew from the trial voluntarily; therefore, 477 patients were administered study drug. One patient in the propofol group withdrew from the study voluntarily after receiving propofol. Finally, 357 patients randomized to receive remimazolam besylate and 119 patients randomized to receive propofol were included in the analyses.

Patients at 10 different centers were enrolled in this trial. The number of enrolled patients at each center ranged from 16–72 (Supplementary Table S1).

### Baseline demographic and clinical characteristics

The pre-treatment demographic and clinical characteristics of the patients randomized to receive remimazolam besylate or propofol were well balanced (Table 2).

**TABLE 2:** Baseline characteristics of study patients.

Characteristic	Remimazolam	Propofol	*p* value
Age (year, Q1∼Q3)	44.3 (33.0–54.0)	46.4 (37.5–56.0)	0.095
BMI (kg/m^2^, Q1∼Q3)	22.82 (20.70–24.90)	22.87 (21.10–24.60)	0.843
Male, n (%)	154 (43.1)	56 (46.7)	0.500
Heart rate (beats/min, Q1∼Q3)	74.9 (67.0–81.0)	74.4 (67.0–80.5)	0.608
Systolic blood pressure (mmHg, Q1∼Q3)	115.7 (106.0–124.0)	117.2 (107.0–127.0)	0.286
Diastolic blood pressure (mmHg, Q1∼Q3)	74.6 (68.0–82.0)	75.3 (69.0–82.0)	0.542
Respiratory rate (breaths/min, Q1∼Q3)	16.0 (14.0–18.0)	16.3 (14.0–19.0)	0.371
Modified Mallampati Score, Grade I, n (%)	302 (84.6)	105 (87.5)	0.436
ASA grade, Grade I, n (%)	318 (89.1)	99 (82.5)	0.060

### Drug exposure

There was no significant difference in first exposure time to remimazolam besylate or propofol; however, total exposure time was significantly higher for remimazolam besylate compared to propofol (Table 3). There were no significant differences in fentanyl exposure, fentanyl exposure time, or use of rescue medication between the remimazolam group and the propofol group (Table 4, Supplementary Table S2).

**TABLE 3:** Study drug exposure.

	Remimazolam	Propofol	*p* value
First exposure time (s, Q1∼Q3)	59.9 (60.0–60.0)	59.9 (60.0–60.0)	0.959
Initial exposure (mg, Q1∼Q3)	7.00 (7.00–7.00)	92.09 (81.00–104.00)	—
Top-up dose n (%)	—	—	—
Yes	247 (69.2)	65 (54.2)	0.003
No	110 (30.8)	55 (45.8)	—
Number of top-up doses, n (%)			
0	110 (30.8)	55 (45.8)	*p* = 0.008
1	152 (42.6)	49 (40.8)	—
2	61 (17.1)	10 (8.3)	—
3	26 (7.3)	3 (2.5)	—
4	5 (1.4)	3 (2.5)	—
5	3 (0.8)	0 (0)	—
≤5 doses, n (%)			
Yes	357 (100.0)	120 (100.0)	NA
No	0 (0)	0 (0)	—
Total exposure time (s, Q1∼Q3)	76.4 (60.0–90.0)	71.1 (60.0–75.0)	<0.001
Total exposure (mg, Q1∼Q3)	9.69 (7.00–12.00)	114.45 (92.00–130.00)	<0.001

First exposure time = end time of first dose- start time of first dose.

Total exposure time = (end time of first dose–start time of first dose) + ∑ (end time of additional dose–start time of additional dose).

Total exposure = first exposure + additional total exposure.

**TABLE 4:** Fentanyl exposure and rescue medication.

Characteristic	Remimazolam	Propofol	*p* value
Fentanyl exposure (μg, Q1∼Q3)	49.6 (50.0–50.0)	49.3 (50.0–50.0)	0.275
Fentanyl exposure time (s, Q1∼Q3)	29.0 (25.0–30.0)	28.6 (24.5–30.0)	0.639
Rescue medication, n (%)			
Yes	4 (1.1)	1 (0.8)	0.789
No	353 (98.9)	119 (99.2)	—
Frequency of remediation time			
0	353 (98.9)	119 (99.2)	*p* = 0.845
1	3 (0.8)	1 (0.8)	—
2	1 (0.3)	0 (0)	—

Fentanyl exposure time = end time of fentanyl administration- start time of fentanyl administration.

### Safety

A total of 242 (67.8%) patients administered remimazolam besylate experienced 532 adverse events, and 101 (84.2%) patients administered propofol experienced 250 adverse events (Supplementary Table S3). The incidence of adverse events was significantly higher in patients administered propofol compared to patients administered remimazolam besylate (*p* = 0.001; Table 5). There were no serious adverse events or deaths in this study.

**TABLE 5:** Incidence of major adverse events.

	Remimazolam n (%)	Propofol n (%)	*p* value
Oxygen Desaturation (during the procedure)			
Yes	4 (1.1)	8 (6.7)	0.001
No	353 (98.9)	112 (93.3)	—
Hypotension (during the procedure)			
Yes	38 (10.6)	35 (29.2)	<0.0001
No	319 (89.4)	85 (70.8)	—
Hypotension requiring intervention (during the procedure)			
Yes	2 (0.6)	2 (1.7)	0.250
No	355 (99.4)	118 (98.3)	—
Injection pain (during the procedure)			
Yes	8 (2.3)	42 (35.3)	<0.0001
No	347 (97.7)	77 (64.7)	—
Elevated alanine aminotransferase (after the procedure)			
Yes	5 (1.4)	8 (6.7)	0.002
No	352 (98.6)	112 (93.3)	—
Adverse events			
Yes	242 (67.8)	101 (84.2)	0.001
No	115 (32.2)	19 (15.8)	—
Severe adverse events			
Yes	0 (0)	0 (0)	NA
No	357 (100.0)	120 (100.0)	—

Adverse events experienced by >5% of patients administered remimazolam besylate included dizziness (36.4%), gait disorder (24.4%), elevated blood bilirubin (11.8%), and hypotension (10.9%). Adverse events experienced by >5% of patients administered propofol included pain at the injection site (35.0%), hypotension (31.7%), dizziness (26.7%), gait disorder (21.7%), elevated blood bilirubin (14.2%), bradycardia (10.0%), oxygen desaturation (6.7%), and elevated ALT (6.7%).

Among the clinically important adverse events of special interest, 4 (1.1%) patients administered remimazolam besylate and 8 (6.7%) patients administered propofol experienced oxygen desaturation. The incidence of oxygen desaturation was significantly higher in patients administered propofol compared to patients administered remimazolam besylate (*p* = 0.001, Table 5). A total of 38 (10.6%) patients administered remimazolam besylate and 35 (29.2%) patients administered propofol experienced hypotension. The incidence of hypotension was significantly higher in patients administered propofol compared to patients administered remimazolam besylate (*p* < 0.0001, Table 5). A total of 2 (0.6%) patients administered remimazolam besylate and 2 (1.7%) patients administered propofol experienced hypotension requiring intervention, and there was no significant difference between the remimazolam group and the propofol group (*p* = 0.250, Table 5). In addition, the proportion of patients with injection pain was significantly lower in patients administered remimazolam besylate compared to patients administered propofol (*p* < 0.0001, Table 5). The incidence of elevated ALT was significantly higher in patients administered propofol compared to patients administered remimazolam besylate (6.7% vs. 1.4%, *p* = 0.002, Table 5).

At the follow-up visit, laboratory examinations showed 7% of patients administered remimazolam besylate and 10% of patients administered propofol had elevated blood bilirubin. <5% of patients administered remimazolam besylate or propofol had other liver and kidney function-related adverse events, and all events were mild.

### Efficacy

In the FAS, sedation success rates were 98.9% and 99.2% in patients administered remimazolam besylate or propofol, respectively. In the PPS, sedation success rates were 99.4% and 99.2% in patients administered remimazolam besylate or propofol, respectively. There were no significant differences between the remimazolam group and the propofol group. After adjusting for centers, ASA stratification and gender, the 95% CI for the difference in sedation success rates between patients administered remimazolam besylate or propofol was -2.5%–2.3%, while the value before adjustment was -2.1%–3.5%. According to the predetermined noninferiority margin of -5%, the success rate of sedation in patients administered remimazolam besylate can be considered non-inferior to the success rate of sedation in patients administered propofol.

Among the successfully sedated patients, mean time from the start of study drug administration to MOAA/S score ≤3 was significantly longer in patients administered remimazolam besylate (1.45 min) compared to patients administered propofol (1.24 min) (*p* < 0.001, Table 6). Mean time at minimum MOAA/S score prior to first top-up of study drug was significantly longer for patients administered remimazolam besylate (1.73 min) than for patients administered propofol (1.48 min) (*p* < 0.001; Table 7). Mean minimum MOAA/S score after first administration of study drug and prior to first top-up of study medication was significantly higher in patients administered remimazolam besylate (0.5) compared to patients administered propofol (0.2) (*p* < 0.001; Table 7).

**TABLE 6:** Time (min) from the start of administration of study drug to MOAA/S score ≤3 in successfully sedated patients.

	Remimazolam n (%)	Propofol n (%)	*p* value
N	353	119	<0.001
Median	1.50	1.00	—
Q1∼Q3	1.00–1.50	1.00–1.50	—
Min∼Max	1–6	1–4	—
Mean	1.45	1.24	—

**TABLE 7:** Minimum MOAA/S score after first administration of study drug and prior to first top-up of study drug.

	Remimazolam	Propofol	*p* value
MOAA/S minimum value			
Mean ± SD	0.5 ± 1.09	0.2 ± 0.68	<0.001
95%CI	0.4–0.7	0.0–0.3	—
Min∼Max	0–4	0–4	—
Median	0	0	—
Time at MOAA/S minimum value (min)			
Mean ± SD	1.73 ± 0.536	1.48 ± 0.476	<0.001
95%CI	1.67–1.78	1.40–1.57	—
Min∼Max	1–4	1–3	—
Median	1.50	1.50	—

Mean time from the end of the last administration of study drug to fully alert was not significantly different between the remimazolam group and the propofol group (*p* = 0.339, Table 8). However, among the successfully sedated patients, mean time from the end of the last administration of study drug to discharge was significantly shorter (20.3 min) for patients administered remimazolam besylate than for patients administered propofol (21.8 min) (*p* = 0.020, Table 8).

**TABLE 8:** Time from the end of the last administration of study drug to fully alert and discharge in successfully sedated patients.

	Remimazolam	Propofol	*p* value
Time from the end of the last administration to fully alert (min)			
Median	7.570	7.420	0.339
Q1∼Q3	6.015–9.000	5.830–9.000	—
Min∼Max	1.55–21.83	2.45–15	—
Mean	7.825	7.590	—
Time from the end of the last administration to discharge (min)			
Median	18.420	20.000	0.020
Q1∼Q3	15.420–23.200	16.330–26.000	—
Min∼Max	9.98–137.38	10.08–46.83	—
Mean	20.312	21.795	—

**FIGURE 2:**
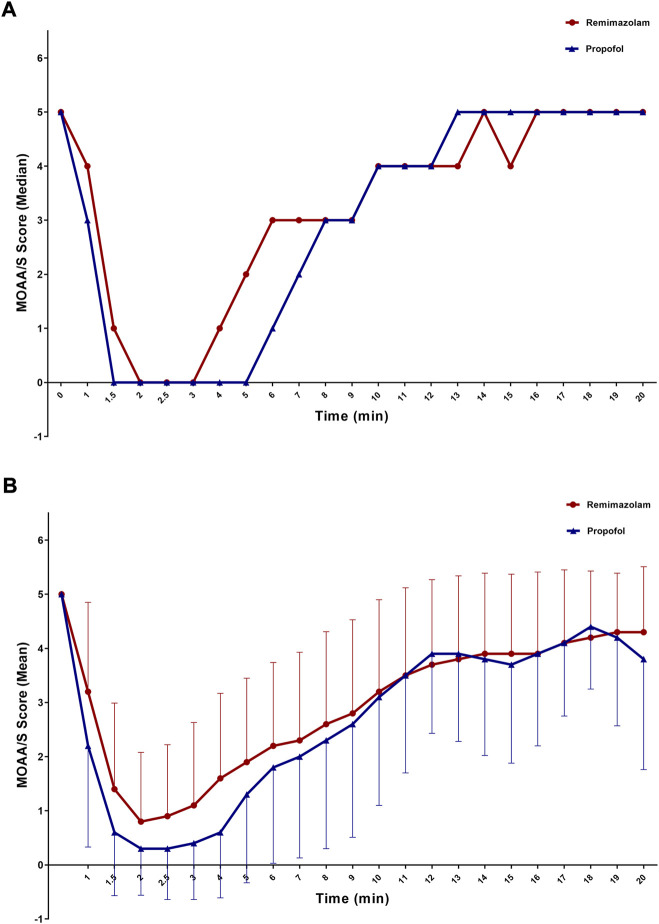
MOAA/S score over time **(A)** Median; **(B)** Mean; FAS). MOAA/S score after using rescue drugs was not included.

Changes in median/mean MOAA/S score for the FAS over time are shown in Figures 2A,B and Supplementary Table S4. After study drug administration, median/mean MOAA/S scores in patients administered remimazolam besylate or propofol declined. The decline in median/mean MOAA/S score was greater in patients administered propofol compared to remimazolam besylate. Median minimum MOAA/S score after study drug administration was reached at 2 min and 1.5 min in patients administered remimazolam besylate or propofol, respectively. Median MOAA/S score in patients administered remimazolam besylate began to rise 4 min after study drug administration, and recovered to a median MOAA/S score of 5 at 16 min after study drug administration. Median MOAA/S score in patients administered propofol began to rise 6 min after study drug administration, and recovered to a median MOAA/S score of 5 at 13 min after study drug administration (Figure 2A; Supplementary Table S4).

## Discussion

This Phase Ⅲ clinical trial evaluated the safety and efficacy of remimazolam besylate vs. propofol injection for sedation in patients undergoing a diagnostic or therapeutic colonoscopy in China. Findings showed that remimazolam besylate had a better safety and tolerability profile and similar sedative efficacy to propofol in this setting, suggesting that remimazolam besylate has potential as a sedative agent for colonoscopy.

In this trial, remimazolam besylate and propofol induction doses were according to product monographs and expert consensus on endoscopic anesthesia in China. Doses were safe and well tolerated. There were no serious adverse events that led to study discontinuation in the remimazolam group or the propofol group. The incidence of all adverse events and clinically important adverse events of special interest (oxygen desaturation and/or hypotension during sedation) were significantly lower in patients administered remimazolam besylate compared to propofol, implying a more beneficial safety profile for remimazolam besylate. At the follow-up visit, the results of laboratory examinations showed that 7% of patients administered remimazolam besylate and 10% of patients administered propofol experienced elevated blood bilirubin, while <5% of patients administered remimazolam besylate or propofol had other liver and kidney function-related adverse events, and all events were mild. These findings indicate that remimazolam besylate and propofol had no significant effects on liver and/or kidney function.

Efficacy evaluations showed no significant difference in the sedation success rate in patients administered remimazolam besylate or propofol; propofol had a significantly shorter onset of action than remimazolam, but the difference was not considered clinically significant; and propofol achieved a deeper level of sedation and the minimal MOAA/S score was maintained for a longer time. There was no significant difference in mean time to fully alert after the end of the last administration of study drug, but mean time to discharge after the end of the last administration of study drug was significantly shorter for patients administered remimazolam besylate. Diagnostic or therapeutic colonoscopy does not usually exceed 30 min. The results of the present study indicate that remimazolam besylate and propofol effectively achieved and maintained an adequate level of sedation for the procedure; however, the use of remimazolam besylate appeared to avoid the deep sedation that often occurred in propofol sedation. The proportion of patients with injection pain was significantly lower in patients administered remimazolam besylate compared to patients administered propofol. Pain after injection is one of the most common adverse effects of propofol sedation. Findings from this study suggest that remimazolam besylate may avoid propofol injection pain while achieving the same sedative effect in diagnostic or therapeutic colonoscopy.

Previous studies have investigated the efficacy and safety of remimazolam for procedural sedation and analgesia in patients undergoing colonoscopy, upper gastrointestinal endoscopy or bronchoscopy, and in surgical patients receiving general anesthesia (Chen et al., 2021). Clinical administration of remimazolam has mostly been studied in diagnostic colonoscopy (Worthington et al., 2013; Pambianco et al., 2016; Rex et al., 2018; Chen et al., 2020; Rex et al., 2021). In a Phase 1b clinical trial of healthy volunteers undergoing colonoscopy, remimazolam (0.04, 0.075, 0.1 mg/kg as initial dose, with 0.04 mg/kg top-up doses) combined with low-dose fentanyl was not associated with serious adverse events and had a successful sedation rate of 77% with onset of sedation <1 min. Median time to fully alert ranged from 7–9 min (Worthington et al., 2013). In a Phase IIb clinical trial of patients undergoing colonoscopy, remimazolam (8.0, 7.0, 5.0 mg as initial dose, with 3.0, 2.0, 3.0 mg top-up doses) was superior to midazolam (2.5 mg as initial dose, with 1 mg top-up dose) for proving adequate sedation with a high procedural success rate (>92% vs. 75%; *p* = 0.007) (Pambianco et al., 2016). In a Phase III clinical trial of patients undergoing colonoscopy, mean recovery time to fully alert was 7.35, 21.95, and 15.84 min and procedural success rates were 91.3%, 1.7%, and 25.2% for remimazolam (5.0 mg as initial dose, with 2.5 mg top-up dose), placebo, and midazolam (1.0/0.5 mg - 1.75/1.0 mg) (Rex et al., 2018), respectively. In a Phase III clinical trial of ASA physical status III/IV patients undergoing high-risk colonoscopy, mean recovery time to fully alert was 3.0, 5.3, and 7.0 min and procedural success rates were 87.1%, 0.0%, and 13.3% for remimazolam (2.5–5.0 mg as initial dose, with 1.25–2.5 mg top-up doses), placebo, and midazolam (1.0 mg as initial dose, with 0.5 mg top-up dose) (Rex et al., 2021), respectively. In another Phase III trial of patients undergoing colonoscopy in China, mean recovery time to fully alert was 8.17 and 7.74 min and the procedural success rates were 96.91% and 100% for remimazolam (5.0 mg as initial dose, with 2.5 mg top-up dose) and propofol (1.5 mg/kg as initial dose, with 0.5 mg/kg top-up dose), and remimazolam was considered non-inferior to propofol in sedative efficacy (Chen et al., 2020).

Taken together, findings from these previous clinical trials and the present study confirm that remimazolam has a similar safety and efficacy profile to midazolam or propofol for providing sedation in colonoscopy. However, results should be interpreted with caution, as the doses of remimazolam and comparator varied among different clinical trials, pharmacologic data reporting equipotent doses of remimazolam compared to other sedatives are scarce, and no studies have investigated the quality of sedation and recovery with remimazolam (Chen et al., 2021).

The present study was associated with several limitations. First, due to the single-blind nature of the study, data collection and clinical decision-making were mainly performed by unblinded individuals, which may have caused selection bias. Second, this study did not consider whether the increased dose of remimazolam will increase the incidence of adverse events (oxygen desaturation, circulatory instability, etc.) when the MOAA/S score was ≤1. The trial focused on whether colonoscopy can be successfully completed, and used this as the main efficacy indicator, ignoring the difference in MOAA/S scores between the patients administered remimazolam and propofol. Further studies are needed to explore the association between increased remimazolam dose and the incidence of adverse events. Third, patients enrolled in this trial were aged 18–65 years; therefore, the safety and efficacy of remimazolam besylate for sedation in younger or elderly patients undergoing a diagnostic or therapeutic colonoscopy is unknown. Fourth, data on the effectiveness of colonoscopy were not collected. Further information on procedure time, recovery time, or time to reach the cecum are required to assess the quality of colonoscopy associated with adequate sedation. Finally, all the participating centers were top-ranked hospitals; therefore, the results may not be generalizable to other centers due to differences in healthcare providers’ technical skills.

## Conclusion

Remimazolam besylate had a better safety and tolerability profile and similar sedative efficacy to propofol in patients undergoing a diagnostic or therapeutic colonoscopy in China, suggesting that remimazolam besylate has potential as a sedative agent for colonoscopy.

## Data Availability

The original contributions presented in the study are included in the article/Supplementary Material, further inquiries can be directed to the corresponding authors.

## Abbreviations

ASA, American Society of Anesthesiologists; ALT, Alanine aminotransferase; AST, Aspartate transaminase; BMI, Body mass index; FAS, Full analysis set; GABAA, γ-aminobutyric acid A; ICU, Intensive care unit; PPS, Per-protocol set; SS, Safety set; SBP, Systolic blood pressure; ULN, Upper limit of normal.
