# Supplementary material for: Safety and efficacy of remimazolam besylate in patients undergoing colonoscopy: A multicentre, single-blind, randomized, controlled, phase Ⅲ trial
Source: Front Pharmacol. 2022 Oct 5;13:900723. doi: 10.3389/fphar.2022.900723 (PMC9579314; doi:10.3389/fphar.2022.900723)
Supplement: Supplementary file 1 [file DataSheet1.docx]

**Supplementary Table 1 Patient distribution**

| Centers | Enrollment (Remimazolam/ Propofol) | FAS | PPS | SS |
| --- | --- | --- | --- | --- |
| Total | 480 (360/120) | 477 | 473 | 477 |
| Xiangya Hospital Central South University | 72 (54/18) | 72 | 71 | 72 |
| Third Xiangya Hospital of Central South University | 72 (54/18) | 72 | 72 | 72 |
| The Second Affiliated Hospital of Wenzhou Medical University | 48 (36/12) | 48 | 46 | 48 |
| The First Affiliated Hospital of Zhengzhou University | 40 (30/10) | 39 | 39 | 39 |
| The Second Affiliated Hospital of Nanchang University | 72 (54/18) | 72 | 72 | 72 |
| Peking University International Hospital | 16 (12/4) | 16 | 16 | 16 |
| Wuhan Puai Hospital | 32 (24/8) | 32 | 31 | 32 |
| Affiliated Hospital of Zunyi Medical College | 64 (48/16) | 63 | 63 | 63 |
| The First Affiliated Hospital of Xi 'an Jiaotong University | 24 (18/6) | 24 | 24 | 24 |
| Affiliated Hospital of Guizhou Medical University | 40 (30/10) | 39 | 39 | 39 |

FAS: Full analysis set; PPS: Per-protocol set; SS: Safety set

**Supplementary Table 2 Proportional analysis of rescue medication (stratified by centers) (FAS)**

| **Centers** | **Rescue medication** | **Remimazolam**  **n (%)** | **Propofol**  **n (%)** |
| --- | --- | --- | --- |
| Total | Yes | 4 (1.1) | 1 (0.8) |
|  | No | 353 (98.9) | 119 (99.2) |
|  |  |  |  |
| Xiangya Hospital Central South University | Yes | 0 (0) | 0 (0) |
|  | No | 54 (100.0) | 18 (100.0) |
|  |  |  |  |
|  |  |  |  |
| Third Xiangya Hospital of Central South University | Yes | 0 (0) | 1 (5.6) |
|  | No | 54 (100.0) | 17 (94.4) |
|  |  |  |  |
|  |  |  |  |
| The Second Affiliated Hospital of Wenzhou Medical University | Yes | 3 (8.3) | 0 (0) |
|  | No | 33 (91.7) | 12 (100.0) |
|  |  |  |  |
| The First Affiliated Hospital of Zhengzhou University | Yes | 1 (3.4) | 0 (0) |
|  | No | 28 (96.6) | 10 (100.0) |
|  |  |  |  |
|  |  |  |  |
| The Second Affiliated Hospital of Nanchang University | Yes | 0 (0) | 0 (0) |
|  | No | 54 (100.0) | 18 (100.0) |
|  |  |  |  |
| Peking University International Hospital | Yes | 0 (0) | 0 (0) |
|  | No | 12 (100.0) | 4 (100.0) |
|  |  |  |  |
| Wuhan Puai Hospital | Yes | 0 (0) | 0 (0) |
|  | No | 24 (100.0) | 8 (100.0) |
|  |  |  |  |
|  |  |  |  |
| Affiliated Hospital of Zunyi Medical College | Yes | 0 (0) | 0 (0) |
|  | No | 47 (100.0) | 16 (100.0) |
|  |  |  |  |
|  |  |  |  |
| The First Affiliated Hospital of Xi 'an Jiaotong University | Yes | 0 (0) | 0 (0) |
|  | No | 18 (100.0) | 6 (100.0) |
|  |  |  |  |
|  |  |  |  |
| Affiliated Hospital of Guizhou Medical University | Yes | 0 (0) | 0 (0) |
|  | No | 29 (100.0) | 10 (100.0) |

**Supplementary Table 3 Adverse events (stratified by centers)**

| **Centers** | **Adverse events** | **Remimazolam**  **n (%)** | **Propofol**  **n (%)** |
| --- | --- | --- | --- |
| Total | Yes | 242 (67.8) | 101 (84.2) |
|  | No | 115 (32.2) | 19 (15.8) |
|  |  |  |  |
| Xiangya Hospital Central South University | Yes | 46 (85.2) | 16 (88.9) |
|  | No | 8 (14.8) | 2 (11.1) |
|  |  |  |  |
|  |  |  |  |
| Third Xiangya Hospital of Central South University | Yes | 28 (51.9) | 15(83.3) |
|  | No | 26 (48.1) | 3(16.7) |
|  |  |  |  |
|  |  |  |  |
| The Second Affiliated Hospital of Wenzhou Medical University | Yes | 33 (91.7) | 11 (91.7) |
|  | No | 3 (8.3) | 1 (8.3) |
|  |  |  |  |
| The First Affiliated Hospital of Zhengzhou University | Yes | 25(86.2) | 9 (90.0) |
|  | No | 4 (13.8) | 1 (10.0) |
|  |  |  |  |
|  |  |  |  |
| The Second Affiliated Hospital of Nanchang University | Yes | 31 (57.4) | 14 (77.8) |
|  | No | 23 (42.6) | 4 (22.2) |
|  |  |  |  |
| Peking University International Hospital | Yes | 7 (58.3) | 3 (75.0) |
|  | No | 5 (41.7) | 1 (25.0) |
|  |  |  |  |
| Wuhan Puai Hospital | Yes | 18(75.0) | 7 (87.5) |
|  | No | 6(25.0) | 1 (12.5) |
|  |  |  |  |
|  |  |  |  |
| Affiliated Hospital of Zunyi Medical College | Yes | 20 (42.6) | 14 (87.5) |
|  | No | 27 (57.4) | 2 (12.5) |
|  |  |  |  |
|  |  |  |  |
| The First Affiliated Hospital of Xi 'an Jiaotong University | Yes | 11 (61.1) | 3 (50.0) |
|  | No | 7 (38.9) | 3 (50.0) |
|  |  |  |  |
|  |  |  |  |
| Affiliated Hospital of Guizhou Medical University | Yes | 23 (79.3) | 9 (90.0) |
|  | No | 6 (20.7) | 1 (10.0) |

**Supplementary Table 4 Change in MOAA/S score over time**

|  | **Remimazolam** | **Propofol** | **p value** |
| --- | --- | --- | --- |
| Baseline |  |  | NA |
| N | 357 | 120 |  |
| Mean±SD | 5.0±0 | 5.0±0 |  |
| 95%CI | NA～NA | NA～NA |  |
| Min～Max | 5～5 | 5～5 |  |
| Median | 5 | 5 |  |
| 1 min after the first administration |  |  | <0.001 |
| N | 357 | 120 |  |
| Mean±SD | 3.2±1.65 | 2.2±1.87 |  |
| 95%CI | 3.1～3.4 | 1.8～2.5 |  |
| Min～Max | 0～5 | 0～5 |  |
| Median | 4 | 3 |  |
| 1.5 min after the first administration |  |  | <0.001 |
| N | 357 | 120 |  |
| Mean±SD | 1.4±1.59 | 0.6±1.17 |  |
| 95%CI | 1.2～1.6 | 0.4～0.8 |  |
| Min～Max | 0～5 | 0～4 |  |
| Median | 1 | 0 |  |
| 2 min after the first administration |  |  | <0.001 |
| N | 357 | 120 |  |
| Mean±SD | 0.8±1.28 | 0.3±0.86 |  |
| 95%CI | 0.7～0.9 | 0.1～0.4 |  |
| Min～Max | 0～4 | 0～4 |  |
| Median | 0 | 0 |  |
| 2.5 min after the first administration |  |  | <0.001 |
| N | 357 | 120 |  |
| Mean±SD | 0.9±1.32 | 0.3±0.94 |  |
| 95%CI | 0.7～1.0 | 0.1～0.4 |  |
| Min～Max | 0～5 | 0～5 |  |
| Median | 0 | 0 |  |
| 3 min after the first administration |  |  | <0.001 |
| N | 357 | 120 |  |
| Mean±SD | 1.1±1.53 | 0.4±1.04 |  |
| 95%CI | 1.0～1.3 | 0.2～0.5 |  |
| Min～Max | 0～5 | 0～5 |  |
| Median | 0 | 0 |  |
| 4 min after the first administration |  |  | <0.001 |
| N | 357 | 120 |  |
| Mean±SD | 1.6±1.57 | 0.6±1.21 |  |
| 95%CI | 1.4～1.7 | 0.3～0.8 |  |
| Min～Max | 0～5 | 0～5 |  |
| Median | 1 | 0 |  |
| 5 min after the first administration |  |  | <0.001 |
| N | 357 | 120 |  |
| Mean±SD | 1.9±1.55 | 1.3±1.63 |  |
| 95%CI | 1.8～2.1 | 1.0～1.6 |  |
| Min～Max | 0～5 | 0～5 |  |
| Median | 2 | 0 |  |
| 6 min after the first administration |  |  | 0.004 |
| N | 357 | 120 |  |
| Mean±SD | 2.2±1.54 | 1.8±1.77 |  |
| 95%CI | 2.1～2.4 | 1.4～2.1 |  |
| Min～Max | 0～5 | 0～5 |  |
| Median | 3 | 1 |  |
| 7 min after the first administration |  |  | 0.09 |
| N | 357 | 120 |  |
| Mean±SD | 2.3±1.63 | 2.0±1.87 |  |
| 95%CI | 2.2～2.5 | 1.7～2.4 |  |
| Min～Max | 0～5 | 0～5 |  |
| Median | 3 | 2 |  |
| 8 min after the first administration |  |  | 0.087 |
| N | 355 | 120 |  |
| Mean±SD | 2.6±1.71 | 2.3±2.00 |  |
| 95%CI | 2.4～2.8 | 1.9～2.6 |  |
| Min～Max | 0～5 | 0～5 |  |
| Median | 3 | 3 |  |
| 9 min after the first administration |  |  | 0.314 |
| N(NMiss) | 355(0) | 118(2) |  |
| Mean±SD | 2.8±1.73 | 2.6±2.09 |  |
| 95%CI | 2.7～3.0 | 2.2～3.0 |  |
| Min～Max | 0～5 | 0～5 |  |
| Median | 3 | 3 |  |
| 10 min after the first administration |  |  | 0.515 |
| N(NMiss) | 344(10) | 111(9) |  |
| Mean±SD | 3.2±1.70 | 3.1±2.00 |  |
| 95%CI | 3.0～3.4 | 2.7～3.4 |  |
| Min～Max | 0～5 | 0～5 |  |
| Median | 4 | 4 |  |
| 11 min after the first administration |  |  | 0.871 |
| N(NMiss) | 330(24) | 105(15) |  |
| Mean±SD | 3.5±1.62 | 3.5±1.80 |  |
| 95%CI | 3.3～3.7 | 3.2～3.9 |  |
| Min～Max | 0～5 | 0～5 |  |
| Median | 4 | 4 |  |
| 12 min after the first administration |  |  | 0.209 |
| N(NMiss) | 300(54) | 88(32) |  |
| Mean±SD | 3.7±1.57 | 3.9±1.47 |  |
| 95%CI | 3.5～3.9 | 3.6～4.3 |  |
| Min～Max | 0～5 | 0～5 |  |
| Median | 4 | 4 |  |
| 13 min after the first administration |  |  | 0.62 |
| N(NMiss) | 262(92) | 75(45) |  |
| Mean±SD | 3.8±1.54 | 3.9±1.62 |  |
| 95%CI | 3.6～4.0 | 3.5～4.3 |  |
| Min～Max | 0～5 | 0～5 |  |
| Median | 4 | 5 |  |
| 14 min after the first administration |  |  | 0.589 |
| N(NMiss) | 222(132) | 61(59) |  |
| Mean±SD | 3.9±1.49 | 3.8±1.78 |  |
| 95%CI | 3.7～4.1 | 3.4～4.3 |  |
| Min～Max | 0～5 | 0～5 |  |
| Median | 5 | 5 |  |
| 15 min after the first administration |  |  | 0.564 |
| N(NMiss) | 175(179) | 49(70) |  |
| Mean±SD | 3.9±1.47 | 3.7±1.82 |  |
| 95%CI | 3.7～4.1 | 3.2～4.3 |  |
| Min～Max | 0～5 | 0～5 |  |
| Median | 4 | 5 |  |
| 16 min after the first administration |  |  | 0.843 |
| N(NMiss) | 145(209) | 38(81) |  |
| Mean±SD | 3.9±1.51 | 3.9±1.70 |  |
| 95%CI | 3.7～4.2 | 3.3～4.4 |  |
| Min～Max | 0～5 | 0～5 |  |
| Median | 5 | 5 |  |
| 17 min after the first administration |  |  | 0.753 |
| N(NMiss) | 113(241) | 28(91) |  |
| Mean±SD | 4.1±1.35 | 4.1±1.35 |  |
| 95%CI | 3.8～4.3 | 3.6～4.7 |  |
| Min～Max | 0～5 | 0～5 |  |
| Median | 5 | 5 |  |
| 18 min after the first administration |  |  | 0.494 |
| N(NMiss) | 94(260) | 25(94) |  |
| Mean±SD | 4.2±1.23 | 4.4±1.15 |  |
| 95%CI | 4.0～4.5 | 3.9～4.9 |  |
| Min～Max | 0～5 | 0～5 |  |
| Median | 5 | 5 |  |
| 19 min after the first administration |  |  | 0.811 |
| N(NMiss) | 75(279) | 18(101) |  |
| Mean±SD | 4.3±1.09 | 4.2±1.63 |  |
| 95%CI | 4.1～4.6 | 3.4～5.0 |  |
| Min～Max | 0～5 | 0～5 |  |
| Median | 5 | 5 |  |
| 20 min after the first administration |  |  | 0.355 |
| N(NMiss) | 62(292) | 15(104) |  |
| Mean±SD | 4.3±1.21 | 3.8±2.04 |  |
| 95%CI | 4.0～4.6 | 2.7～4.9 |  |
| Min～Max | 0～5 | 0～5 |  |
| Median | 5 | 5 |  |
| 21 min after the first administration |  |  | 0.079 |
| N(NMiss) | 46(308) | 9(110) |  |
| Mean±SD | 4.1±1.55 | 3.0±2.35 |  |
| 95%CI | 3.6～4.6 | 1.2～4.8 |  |
| Min～Max | 0～5 | 0～5 |  |
| Median | 5 | 4 |  |
| 22 min after the first administration |  |  | 0.026 |
| N(NMiss) | 32(322) | 6(113) |  |
| Mean±SD | 4.0±1.52 | 2.3±2.07 |  |
| 95%CI | 3.5～4.5 | 0.2～4.5 |  |
| Min～Max | 0～5 | 0～5 |  |
| Median | 5 | 2.5 |  |
| 23 min after the first administration |  |  | 0.030 |
| N(NMiss) | 25(328) | 5(114) |  |
| Mean±SD | 3.7±1.70 | 1.8±1.79 |  |
| 95%CI | 3.0～4.4 | -0.4～4.0 |  |
| Min～Max | 0～5 | 0～4 |  |
| Median | 4 | 2 |  |
| 24 min after the first administration |  |  | 0.367 |
| N(NMiss) | 19(334) | 5(114) |  |
| Mean±SD | 3.7±1.60 | 3.0±0.71 |  |
| 95%CI | 2.9～4.5 | 2.1～3.9 |  |
| Min～Max | 0～5 | 2～4 |  |
| Median | 4 | 3 |  |
| 25 min after the first administration |  |  | 0.544 |
| N(NMiss) | 16(337) | 5(114) |  |
| Mean±SD | 3.6±1.75 | 3.0±1.87 |  |
| 95%CI | 2.6～4.5 | 0.7～5.3 |  |
| Min～Max | 0～5 | 0～5 |  |
| Median | 4 | 3 |  |
| 26 min after the first administration |  |  | 0.441 |
| N(NMiss) | 15(338) | 5(114) |  |
| Mean±SD | 3.9±1.41 | 4.4±0.89 |  |
| 95%CI | 3.1～4.6 | 3.3～5.5 |  |
| Min～Max | 0～5 | 3～5 |  |
| Median | 4 | 5 |  |
| 27 min after the first administration |  |  | 0.379 |
| N(NMiss) | 12(341) | 5(114) |  |
| Mean±SD | 3.9±1.56 | 4.6±0.89 |  |
| 95%CI | 2.9～4.9 | 3.5～5.7 |  |
| Min～Max | 0～5 | 3～5 |  |
| Median | 4.5 | 5 |  |
| 28 min after the first administration |  |  | 0.377 |
| N(NMiss) | 9(344) | 4(115) |  |
| Mean±SD | 4.0±0.87 | 4.5±1.00 |  |
| 95%CI | 3.3～4.7 | 2.9～6.1 |  |
| Min～Max | 2～5 | 3～5 |  |
| Median | 4 | 5 |  |
| 29 min after the first administration |  |  | 0.726 |
| N(NMiss) | 9(344) | 2(117) |  |
| Mean±SD | 4.2±0.67 | 4.0±1.41 |  |
| 95%CI | 3.7～4.7 | -8.7～16.7 |  |
| Min～Max | 3～5 | 3～5 |  |
| Median | 4 | 4 |  |
| 30 min after the first administration |  |  | NA |
| N(NMiss) | 7(346) | 1(118) |  |
| Mean±SD | 4.3±0.95 | 3.0±NA |  |
| 95%CI | 3.4～5.2 | NA～NA |  |
| Min～Max | 3～5 | 3～3 |  |
| Median | 5 | 3 |  |
